# Evaluating the effects of biogas slurry and biochar as a partial substitute for chemical fertilizers on greenhouse tomato growth, root traits, and yield: a minimum data set approach

**DOI:** 10.3389/fpls.2025.1657694

**Published:** 2025-09-23

**Authors:** Qinglin Sa, Jian Zheng, Yan Wang, Xuqin Fu, You Wu, Aosong Liu

**Affiliations:** ^1^ College of Energy and Power Engineering, Lanzhou University of Technology, Lanzhou, China; ^2^ School of Civil and Hydraulic Engineering, Lanzhou University of Technology, Lanzhou, China; ^3^ Key Laboratory of Multi-supply System with Solar Energy and Biomass, Lanzhou, Gansu, China; ^4^ Collaborative Innovation Center for Supporting Technology of Northwest Low-Carbon Towns, Lanzhou, China

**Keywords:** biogas slurry, biochar, root morphology, growth quality index, tomato yield

## Abstract

Excessive chemical fertilizer application and nutrient-free irrigation have contributed to suboptimal crop performance and declining yields in greenhouse production. This study investigated the effects of biogas slurry combined with biochar as a partial chemical fertilizer substitute on the growth, root traits, and yield of greenhouse tomatoes. Under equal nitrogen, phosphorus, and potassium inputs and irrigation conditions, different biogas slurry replacement ratios were compared, including CF (traditional fertilization control), FR (chemical fertilizers only), BS25 (low biogas slurry ratio), BS50 (moderate biogas slurry ratio), BS75 (high biogas slurry ratio), and BS100 (biogas slurry only), along with their corresponding treatments combined with biochar, namely CF+C, FR+C, BS25+C, BS50+C, BS75+C, and BS100+C. Path analysis was used to explore the causal relationships between the growth quality index (GQI) and the minimum data set (MDS), revealing the dominant factors affecting GQI of greenhouse tomatoes. Results showed that BS75+C had the most pronounced promoting effects on plant height (PH), stem diameter (SD), and root activity (RA), especially during the flowering stage. At this stage, RA significantly increased to 358.94 μg g^−1^ h^−1^ in spring and 355.42 μg g^−1^ h^−1^ in autumn (*P* < 0.05). Leaf area (LA), leaf area ratio (LAR), specific leaf area (SLA), and leaf area index (LAI) exhibited a continuous increasing trend throughout the entire tomato growth period. Significant differences in biomass allocation indicators were observed during the flowering and fruiting stages, including root biomass ratio (RBR), stem biomass ratio (SBR), leaf biomass ratio (LBR), and root-to-shoot ratio (RSR) (*P* < 0.05). The GQI under BS75+C reached 0.669, which was higher than that of the other treatments, and showed a highly significant positive correlation with tomato yield (*P* < 0.05). The reliability of the MDS-based evaluation system was confirmed, indicating that it effectively captured representative information from the total data set (TDS). Path analysis further showed that RA, SD, and RBR were the key factors influencing GQI. Further multiple linear regression analysis indicated that SD (Beta = 0.559) and RA (Beta = 0.369) had significant direct effects on GQI, while RBR mainly regulated GQI formation through indirect pathways. Overall, BS75+C emerged as a sustainable and efficient soil management strategy, capable of simultaneously improving root development, plant growth, and yield (151,341 kg ha^−1^) under greenhouse conditions in arid and semi-arid environments. BS25, however, provided the highest economic benefit (672,361.04 yuan ha^−1^), offering a more cost-effective alternative under current production conditions.

## 1 Introduction

Over recent years, the greenhouse vegetable industry has expanded rapidly worldwide, driven by its synergy with food production, seasonal advantages, and contributions to food security and economic growth. In China, tomato (*Solanum lycopersicum* L.) cultivation accounts for the largest area among greenhouse vegetable crops, covering approximately 600,000 hectares and producing about 4.5 × 10^7^ tonnes annually (Wang et al., 2017). Tomatoes are rich in vitamins, minerals, and antioxidants, providing essential nutrients for human health, while also exhibiting favorable fruit quality, strong stress tolerance, and high economic value (Erba et al., 2013). To meet the increasing consumer demand for both yield and quality, growers have applied large amounts of chemical fertilizers to support intensive tomato production (Ferreira et al., 2022). Although this approach can enhance productivity in the short term, excessive and prolonged application does not necessarily ensure continued improvements in plant growth or fruit quality. One of the major challenges associated with high fertilizer input is the risk of nutrient imbalances, which can arise not only from the overuse of mineral fertilizers but also from the variable nutrient composition of organic amendments (Parent et al., 2020). These imbalances may impair nutrient uptake efficiency and disrupt key physiological processes such as fruit development, sugar accumulation, and antioxidant synthesis (Shah et al., 2017; Zhang et al., 2020). Therefore, reducing chemical fertilizer inputs and optimizing nutrient management are not only essential strategies for promoting the sustainable development of greenhouse tomato production, but also align with circular economy principles. By utilizing organic waste resources, such approaches help optimize input structures, improve soil health, and enhance both the growth quality and yield of crops (Islam et al., 2010), ultimately delivering significant ecological and economic benefits.

Biogas slurry, a liquid byproduct generated during anaerobic digestion, primarily contains partially decomposed organic substances, readily available inorganic nutrients (such as nitrogen, phosphorus, and potassium), and various microbial metabolites (Zheng et al., 2022). Studies have demonstrated that the organic matter contained in biogas slurry facilitates the formation of soil aggregates, improves soil physicochemical properties, and effectively enhances soil fertility (Islam et al., 2010). According to Chai et al. (2023), when pig-derived biogas slurry replaced 75% or more of the chemical nitrogen fertilizer, it significantly improved soil quality and enhanced asparagus crop performance. Conversely, Wu et al. (2013) found that excessive application of biogas slurry may cause environmental contamination of soil and water bodies. For instance, when pig-derived biogas slurry accounted for 43.75%–56.25% of total fertilizer input, optimal yield and quality of rapeseed were achieved. These findings highlight that the optimal substitution ratio of biogas slurry for chemical fertilizer still requires further investigation. In addition, the high-water content and low nutrient concentration of biogas slurry remain key limitations to its effective use in replacing chemical fertilizers. The core issue lies in increasing nutrient and water retention of biogas slurry in the crop root zone. Biochar is a porous, carbon-rich, highly aromatic and recalcitrant solid material produced by pyrolysis of biomass such as agricultural and forestry residues under anaerobic or anoxic conditions at high temperatures, and is characterized by good stability, large specific surface area, and strong adsorption capacity (Maroušek et al., 2019). When applied at rates of 40–80 t ha^−1^, biochar has been shown to increase tomato yields by up to 37.8% (Zheng et al., 2023), while also improving soil quality and promoting root development, increasing root biomass by 32%, root length by 52%, and root tip number by 17% compared to treatments without biochar application (Mukhopadhyay et al., 2023; Xiang et al., 2017). Thus, it remains to be determined whether combining biogas slurry with biochar can enhance nutrient and water retention in the root zone, improve fertilizer substitution efficiency, and promote crop growth quality.

Growth quality index (GQI) has been widely recognized as an objective and practical tool for evaluating crop growth performance and overall plant health across various cropping systems (Bame et al., 2014; Yu et al., 2018). It is typically constructed based on spatial and temporal variability in key plant growth parameters. In this study, a minimum data set (MDS) was identified using principal component analysis (PCA) and norm value calculation methods to establish a GQI-based evaluation model for assessing crop growth quality (Sadiq et al., 2021). To date, GQI has been applied in various field crops such as maize, sunflower, and camellia sinensis to quantify the integrated effects of environmental conditions, nutrient management, and cultivation practices on plant growth and yield formation (Bame et al., 2014; Chang et al., 2023; Liu et al., 2025). However, studies focusing on greenhouse vegetable production systems, particularly high-value crops like tomatoes, remain limited. Moreover, while GQI has provided valuable insights into the evaluation of growth performance, its application in evaluating integrated nutrient management strategies under controlled nutrient and irrigation input conditions has not been fully explored. In particular, the combined use of organic and carbon-rich amendments has emerged as a promising approach for improving soil structure, enhancing nutrient availability, and optimizing crop growth in intensive greenhouse systems (Zheng et al., 2022; Mukhopadhyay et al., 2023). However, studies that directly investigate the effects of combined biogas slurry and biochar applications on GQI formation and identify the key growth indicators contributing to GQI within the MDS framework are still lacking.

In this study, greenhouse tomato was selected as the model crop to investigate the effects of various biogas slurry and biochar substitution rates on tomato growth quality and fruit yield. Based on 18 growth-related indicators, an optimized MDS model was constructed, and the GQI was calculated using linear modeling approaches, allowing for a quantitative assessment of the combined effects of biogas slurry and biochar substitution for chemical fertilizers on growth quality. Path analysis was applied to identify the dominant factors influencing variations in GQI and to determine the optimal ratios of biogas slurry, biochar, and chemical fertilizers. The novelty of this research lies in optimizing the combination of biogas slurry and biochar to substitute chemical fertilizers, which effectively improves tomato growth quality and enhances crop yield. This study systematically evaluates the combined impacts of various biogas slurry and biochar substitution rates on tomato growth quality. Using MDS models and path analysis, key factors such as root activity, stem diameter, and root-to-shoot biomass ratio were identified as crucial contributors to the improvement of growth quality. This research integrates growth quality assessments and crop yield under uniform nutrient and irrigation conditions, further enriching the evaluation system for growth quality. The findings provide practical guidance for agricultural management in arid and semi-arid regions of China, offering an effective management model to optimize nutrient management and enhance the sustainable development of protected vegetable production systems.

## 2 Materials and methods

### 2.1 Experimental site overview

The experiment was carried out between March and December 2024 at the Qianglong Greenhouse Demonstration Base in Gouyashan, Qilihe District, Lanzhou City, Gansu Province, China (36.04° N, 103.71° E). Based on international soil texture classification standards, soil texture at the experimental site is silty loam. Before the experiment, soil in the plow layer had bulk density of 1.28 g cm^−3^, initial moisture content of 19.93%, electrical conductivity of 302 µS cm^−1^, and pH of 7.61. Soil organic carbon was 9.81 g kg^−1^, soil total nitrogen was 0.85 g kg^−1^, soil alkaline hydrolyzable nitrogen content was 58.74 mg kg^−1^, soil available potassium was 189.68 mg kg^−1^, soil NH_4_^+^-N content was 2.87 mg kg^−1^, soil NO_3_^−^-N content was 30.93 mg kg^−1^, soil total phosphorus was 2.02 g kg^−1^, and soil available phosphorus was 37.17 mg kg^−1^.

### 2.2 Experimental materials

Tomato seedlings (Fenyan 734, Shandong Chenghao Agricultural Technology Co., Ltd.) were transplanted to the greenhouse when three true leaves were fully expanded. Each plant received a total of 7000 mL of water, applied evenly across a seven-day recovery phase. The formal experiment was initiated immediately following the completion of this recovery period.

The biogas slurry used in this study was obtained from Lanzhou Xinsu Ecological Energy Co., Ltd., where the anaerobic digestion system primarily utilizes vegetable residues as the main inoculum source. Prior to application, the raw slurry was left to settle for approximately two months to allow stabilization of its physicochemical properties. It was then subjected to solid–liquid separation, followed by filtration through a four-layer gauze (32 mesh) to remove coarse suspended particles. The slurry met the safety thresholds for heavy metals in accordance with the Chinese standard GB/T 40750—2021, with total concentrations of zinc (Zn) ≤ 1.33 mg L^–1^, lead (Pb) ≤ 0.01 mg L^–1^, and chromium (Cr) ≤ 0.127 mg L^–1^. In addition, pathogenic bacteria such as Escherichia coli and Salmonella were tested and not detected. Main physicochemical characteristics of the raw biogas slurry were as follows: pH 7.78, moisture content 93.6%, total solids (TS) < 2%, and organic matter content 1.7 g L^–1^. The content of alkaline hydrolyzable nitrogen was 0.888 g L^–1^, total nitrogen was 0.956 g L^–1^, total phosphorus was 0.054 g L^–1^, and total potassium was 0.229 g L^–1^. In addition, the content of plant-available phosphorus was 0.99 mg L^–1^, ammonium nitrogen (NH_4_^+^-N) was 146 mg L^–1^, and nitrate nitrogen (NO_3_^−^-N) was 31.14 mg L^–1^.

The biochar used in this study was supplied by Liaoning Jinhefu Agricultural Development Co., Ltd. It was a high-efficiency, environmentally friendly, carbon-based slow-release fertilizer in powdered form. The raw materials included corn straw, corn cobs, and peanut shells. Biochar production was carried out under low-oxygen conditions, with pyrolysis temperatures ranging from 400 to 600 °C and durations of 4 to 6 hours. According to the Chinese standard for organic fertilizers (NY 525—2021), the contents of heavy metals in the biochar did not exceed the permissible limits, and polycyclic aromatic hydrocarbons (PAHs) were not detected. Main physicochemical properties of the biochar were as follows: total carbon content was 48.19%, primarily in the form of organic carbon (≈ 48.19%, with inorganic carbon negligible), including 38.55% recalcitrant carbon and 9.64% labile carbon; nitrogen content was 0.80%, with a C/N ratio of 60.24%. The biochar also contained 2% trace elements such as zinc, boron, and molybdenum. Other characteristics included a moisture content of 28.36%, pH 9.04, organic matter content 925.74 g kg^–1^, alkaline hydrolyzable nitrogen content 0.159 g kg^–1^, available phosphorus content 0.054 g kg^–1^, and available potassium content 0.384 g kg^–1^. Prior to tomato planting, the biochar was evenly applied to the soil surface and thoroughly incorporated into the 0–30 cm plough layer using a rotary tiller.

Urea (N content 46%) used in this study was supplied by Yunnan Yuntianhua Co., Ltd. (China), calcium superphosphate (P_2_O_5_ content 12%) was provided by Meile Fertilizer Co., Ltd. (Jiangsu, China), and potassium sulfate (K_2_O content 52%) was sourced from Yaran Biotechnology Co., Ltd. (China).

### 2.3 Experimental design

A target fruit yield of 135,000 kg ha^–1^, considered high for the region, was established for the experiment. Total nutrient requirements for the entire tomato growing season were calculated using the nutrient balance method (soil testing recommendations), resulting in an N-P_2_O_5_-K_2_O application rate of 380-180-500 kg ha^–1^ (Sa et al., 2025; Zheng et al., 2024). Nutrients were supplied via biogas slurry, chemical fertilizers, and biochar (**Table 1**).

**Table 1:** Irrigation and fertilizer inputs for each experimental treatment.

Treatments	Total N (kg ha^−1^)			P_2_O_5_ (kg ha^−1^)			K_2_O (kg ha^−1^)			Irrigation volume (m^3^ ha^−1^)	
	Biogas slurry	Biochar	Chemical nitrogen fertilizer	Biogas slurry	Biochar	Chemical phosphate fertilizer	Biogas slurry	Biochar	Chemical potassium fertilizer	Spring 2024	Autumn 2024
CF	0.0	0.0	450.0	0.0	0.0	270.0	0.0	0.0	315.0	1818.9	1359.3
FR	0.0		380.0	0.0		180.0	0.0		500.0	1818.9	1359.3
BS25	95.0		285.0	12.3		167.7	27.5		472.5	1818.9	1359.3
BS50	190.0		190.0	24.6		155.4	55.0		445.0	1818.9	1359.3
BS75	285.0		95.0	36.9		143.1	82.5		417.5	1818.9	1359.3
BS100	380.0		0.0	49.2		130.8	110.0		390.0	1818.9	1359.3
CF+C	0.0	7.2	442.8	0.0	5.6	264.4	0.0	21.0	294.0	1818.9	1359.3
FR+C	0.0	7.2	372.8	0.0	5.6	174.4	0.0	21.0	479.0	1818.9	1359.3
BS25+C	87.8	7.2	285.0	6.7	5.6	167.7	6.5	21.0	472.5	1818.9	1359.3
BS50+C	182.8	7.2	190.0	19.0	5.6	155.4	34.0	21.0	445.0	1818.9	1359.3
BS75+C	277.8	7.2	95.0	31.3	5.6	143.1	61.5	21.0	417.5	1818.9	1359.3
BS100+C	372.8	7.2	0.0	43.6	5.6	130.8	89.0	21.0	390.0	1818.9	1359.3

**Figure 1:**
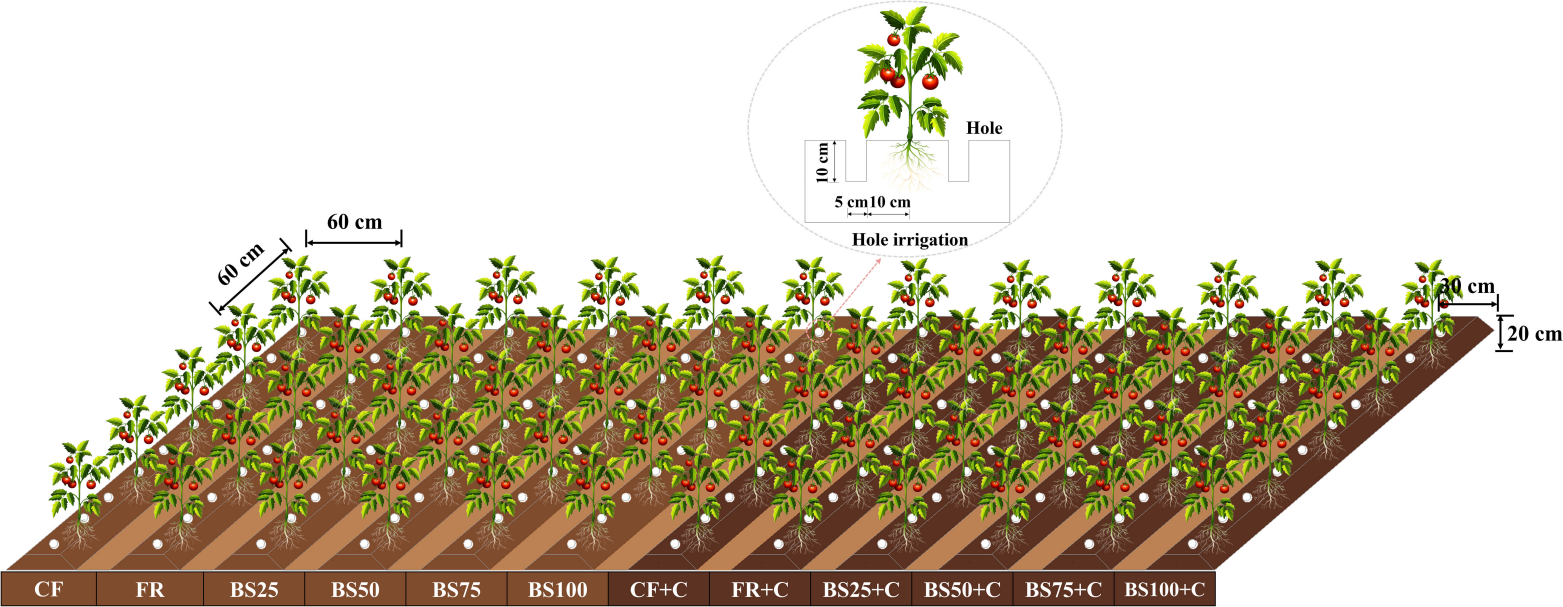
Experimental design diagram.

Each experimental plot was 7.0 m × 6.0 m with 12 ridges. Three identical plots were established. A locally adapted cultivation method involving ridge-furrow planting combined with plastic film mulching was implemented in this study. A single ridge was set in an arched shape (ridge width of 0.3 m, ridge height of 0.2 m, row spacing of 0.6 m, plant spacing of 0.6 m). Each ridge was set as a replicate for the treatment, with a total of 36 ridges in the experiment. A plastic film was laid between ridges to prevent lateral movement of soil water. A planting density of 34,285 plants ha^–1^ was maintained, with 12 plants per ridge. To minimize variability, a randomized complete block design was adopted. Under equal N, P_2_O_5_, and K_2_O nutrient input conditions, with the corresponding biogas slurry-biochar-fertilizer application ratios calculated based on nitrogen content. Total 12 treatments established were as follows (**Figure 1**): CF (traditional fertilization control, 100% nitrogen from chemical fertilizer), FR (chemical fertilizer only, 100% nitrogen from chemical fertilizer based on soil testing recommendations), BS25 (low biogas slurry, 75% nitrogen from chemical fertilizer, 25% from biogas slurry), BS50 (medium biogas slurry, 50% nitrogen from chemical fertilizer, 50% from biogas slurry), BS75 (high biogas slurry, 25% nitrogen from chemical fertilizer, 75% from biogas slurry), BS100 (biogas slurry only), CF+C (conventional fertilization plus biochar, all nitrogen from both chemical fertilizer and biochar), FR+C (chemical fertilizer plus biochar, all nitrogen from both chemical fertilizer and biochar), BS25+C (low biogas slurry plus biochar, 75% nitrogen from chemical fertilizer), BS50+C (medium biogas slurry plus biochar, 50% nitrogen from chemical fertilizer), BS75+C (high biogas slurry plus biochar, 25% nitrogen from chemical fertilizer), and BS100+C (only biogas slurry plus biochar, 0% nitrogen from chemical fertilizer). Each treatment was replicated three plots, and the mean values were used as the final results. Except for the CF and CF+C, all other treatments were designed based on FR (chemical fertilizer only) as the nitrogen reference standard. Biochar was applied as a basal application at a rate of 45 t ha^–1^, as determined by findings from previous studies (Zheng et al., 2023). Specifically, a meta-analysis of field management practices for tomato cultivation indicated that biochar application rates exceeding 40 t ha^–1^, particularly within the range of 40–80 t ha^–1^, could increase yields by up to 37.8%, representing the optimal range for yield enhancement under ideal conditions. Fertilization was distributed as 25%, 15%, 25%, and 35% before transplanting, at the seedling stage, flowering stage, and fruiting stage, respectively, based on the growth characteristics and environmental requirements of tomatoes (Zheng et al., 2024). All other field management practices, including pruning and pest control, were conducted in accordance with locally adopted agronomic standards. For example, the side shoots of tomatoes were periodically managed throughout the entire growth cycle, and shading nets were used for cultivation to control stem-base rot. During the control of whiteflies, a combination of 30% thiamethoxam seed treatment suspension (3000 mL ha^–1^), 250 g L^–1^ pyraclostrobin suspension (1500 mL ha^–1^), and 56% amino acid calcium-magnesium aqueous solution (15,000 mL ha^–1^) was applied through hole irrigation, which also provided control of root rot and other diseases. Additionally, an antiviral agent was sprayed approximately every 10 days to prevent viral infections.

Biogas slurry was delivered using the hole irrigation technique (Zheng et al., 2022). To achieve precise irrigation, holes with a diameter of 5 cm and a depth of 10 cm were positioned on both sides of the ridge, approximately 10 cm away from the plant base. Irrigation was performed every two days. A total of 60 irrigation events were conducted during the 2024 growing season, with the slurry volume uniformly apportioned across all events. Irrigation scheduling was guided by cumulative evaporation data, monitored using a φ20 cm evaporation pan installed at the height of the crop canopy within the greenhouse. Evaporation was recorded at 08:30 AM on each irrigation day, reflecting the cumulative water loss since the previous event. The irrigation requirement for each cycle was computed using Equation 1, from which the volume of biogas slurry applied was subtracted to determine the net water input (Liu et al., 2013). This ensured that each treatment received an identical total irrigation volume.


(1) $$W=K_{p}SE_{p}$$

Where *W* is the irrigation volume (ml), K_p_ denotes the evaporation coefficient (0.8), S indicates the wetted area (1800 cm^2^), and *E_p_* represents the cumulative evaporation recorded by the φ20 cm pan between two successive irrigation intervals (mm).

### 2.4 Sample collection and analysis

#### 2.4.1 Plant height, stem diameter, and yield

In each treatment group, three typical tomato plants were selected and tagged for repeated measurements. Beginning six days post-transplanting and continuing until the topping stage. Plant height was measured with a ruler (accuracy: 1 mm) from the base of the stem to the apex of the plant. Stem diameter was determined using a digital caliper (accuracy: 0.02 mm) by measuring the stem’s cross-sectional area. During fruiting stage, three randomly chosen plants from each treatment were used to measure the weight of individual fruits. Total fruit yield was then calculated by summing the fruit weight across the selected plants.

#### 2.4.2 Leaf area

Leaf area (LA) was estimated using the length-width factor method, while leaf area ratio (LAR), specific leaf area (SLA), leaf area index (LAI), root biomass ratio (RBR), stem biomass ratio (SBR), leaf biomass ratio (LBR), and root-to-shoot ratio (RSR) were determined following the methodology described in reference (Wang et al., 2021). Specifically, SLA is calculated as total leaf area divided by leaf weight; LAR is calculated as total leaf area divided by total plant weight; and LAI is determined by multiplying single plant leaf area by planting density.

#### 2.4.3 Dry matter accumulation

During the seedling stage (spring: March to April; autumn: August to September), flowering stage (spring: April to May; autumn: September to October), and fruiting stage (spring: May to July; autumn: October to December), three plants were randomly chosen under each treatment. Fresh weights of the roots, stems, leaves, and fruits were recorded. Then, each organ was placed in separate bags and oven-dried. Samples were initially heated at 105°C for 60 minutes to deactivate enzymes, followed by drying at 75°C until a constant weight was reached. Subsequently, dry matter mass was recorded.

#### 2.4.4 Root system and root vitality

During the entire tomato cultivation period, three plants exhibiting consistent growth were selected for root analysis. The complete root systems were collected using a comprehensive sampling approach. After thorough washing, the root samples were scanned with a root scanner (Epson Expression 1600 Pro, Japan) and quantified using specialized software (WinRHIZO Pro, Canada) to assess key root characteristics, such as total root length (RL), average root diameter (RAD), root volume (RV), root surface area (RSA), root tip count (RT), branch point number (BN), and root nodule count (RN). Root vitality (RA) was quantified utilizing the triphenyl tetrazolium chloride dehydrogenase assay (Wang et al., 2024).

### 2.5 Growth quality assessment methods

#### 2.5.1 Minimum data set establishment

In this study, a total data set (TDS) consisting of 18 greenhouse tomato growth indicators was compiled to assess crop performance. An important data set (IDS) was derived through Pearson correlation analysis to identify indicators that were significantly associated with tomato yield (*P* < 0.05). The final minimum data set (MDS) was determined using a three-step procedure involving principal component analysis (PCA) and norm value assessment (Sa et al., 2025; Xie et al., 2019; Gong et al., 2015). The norm value for each indicator was calculated using Equation 2:


(2) $$N_{rs}=\sqrt{\sum_{r=1}^{s}\left(U_{rs}^{2}\lambda_{s}\right)}$$

where *N_rs_* indicates the cumulative contribution of the *r* ^-th^ variable across the principal components with eigenvalues greater than 1. *U_rs_* denotes the loading of the *r* ^-th^ variable on the *s* ^-th^ principal component, and *λ_s_* represents the corresponding eigenvalue of the *s* ^-th^ principal component.

#### 2.5.2 Growth quality index calculation

To enable consistent comparison, a linear normalization scheme was adopted to standardize tomato growth parameters on a scale from 0 to 1. Two distinct scoring approaches were employed depending on the characteristics of each indicator: a “more is better” function (Equation 3) and an “optimal range” function (Equation 4) were selected accordingly (Chang et al., 2023).


(3) $$Q_{i}=\frac{V_{i}-V_{min}}{V_{max}-V_{min}}$$

(4) $$Q_{i}=\frac{1}{1+{\left(\frac{V_{i}}{V_{m}}\right)}^{b}}$$

where *Q_i_* denotes the normalized score, *V_i_* is the actual measured value of the indicator, *V_max_* and *V_min_* represent the maximum and minimum values of the indicator, respectively, while *V_m_* is the mean measured value. *b* represents the slope of the equation set at either 2.5 or –2.5 (Mamehpour et al., 2021).

The weight coefficient for each indicator was determined as the ratio of the common factor variance of the individual indicator to the sum of the common factor variances of all indicators. Based on the scoring and weighting of indicators in both the total data set (TDS) and the minimum data set (MDS), the growth quality index (GQI) for greenhouse-grown tomato was derived by integrating factor analysis with a membership function approach, resulting in GQI-TDS and GQI-MDS. A higher GQI value indicated better plant growth quality. The calculation formula is provided in Equation 5 (Bame et al., 2014; Nie et al., 2025).


(5) $$GQI=\sum_{i=1}^{n}W_{i}Q_{i}$$

where *W_i_* denotes the weight assigned to each indicator, *Q_i_* is the normalized score, and *n* refers to the number of indicators included in either the TDS or the MDS.

### 2.6 Path analysis method

Path analysis, built upon correlation and regression analysis, decomposes the correlation coefficients into direct and indirect path coefficients, thereby revealing the relative influence of various factors on the dependent variable and providing a solid foundation for statistical decision-making (Zheng et al., 2023).

### 2.7 Statistical analyses

Data processing was conducted in Microsoft Excel 2018. Graphical visualization, correlation analysis (*P* < 0.05), and linear regression were performed using Origin 2022. Analysis of variance (ANOVA), principal component analysis (PCA), and path analysis were carried out with IBM SPSS Statistics 25. Experimental layout diagram was created using Microsoft PowerPoint.

## 3 Results

### 3.1 Impact of biogas slurry combined with biochar as chemical fertilizer replacement on greenhouse tomato plant growth and fruit yield

#### 3.1.1 Tomato morphological traits and fruit yield

**Figure 2:**
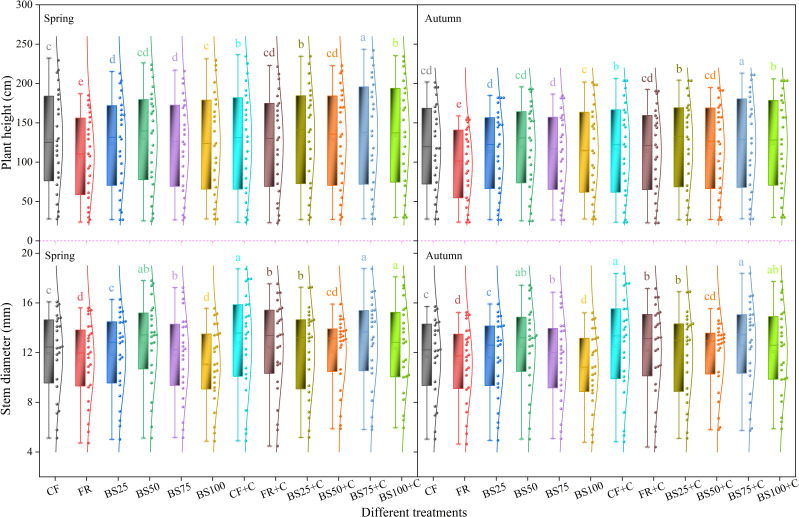
Effects of biogas slurry combined with biochar as chemical fertilizer replacements on plant height and stem diameter of greenhouse tomatoes. Treatments labeled with different letters are significantly different at the 5% level (*P* < 0.05). In the box plots, the solid line and the square inside the box represent the median and the mean, respectively. The lower and upper edges of the box correspond to the first quartile (Q1) and the third quartile (Q3). The whiskers extending from the box indicate 1.5 times the interquartile range (IQR) below Q1 and above Q3, with solid circular points denoting outliers. The curve on the right side of the box depicts the data distribution.

Under equal N-P_2_O_5_-K_2_O input conditions, BS75+C treatment resulted in the highest plant height (243.7 cm in spring and 213.3 cm in autumn), followed by CF+C (236.7 cm in spring and 206.3 cm in autumn), which were 30.30% and 26.56% (spring), and 36.19% and 31.72% (autumn) higher than FR (187.0 cm in spring and 156.6 cm in autumn), respectively (**Figures 2A, C**). Compared to biogas slurry replaced chemical fertilizer treatments, biochar addition significantly increased greenhouse tomato plant height by 1.73%–22.99% (*P* < 0.05). BS75+C treatment had the largest stem diameter, measuring 18.8 mm in spring and 18.4 mm in autumn, followed by CF+C, while FR had the smallest stem diameter (15.4 mm in spring and 15.1 mm in autumn) (**Figures 2B, D**).

**Figure 3:**
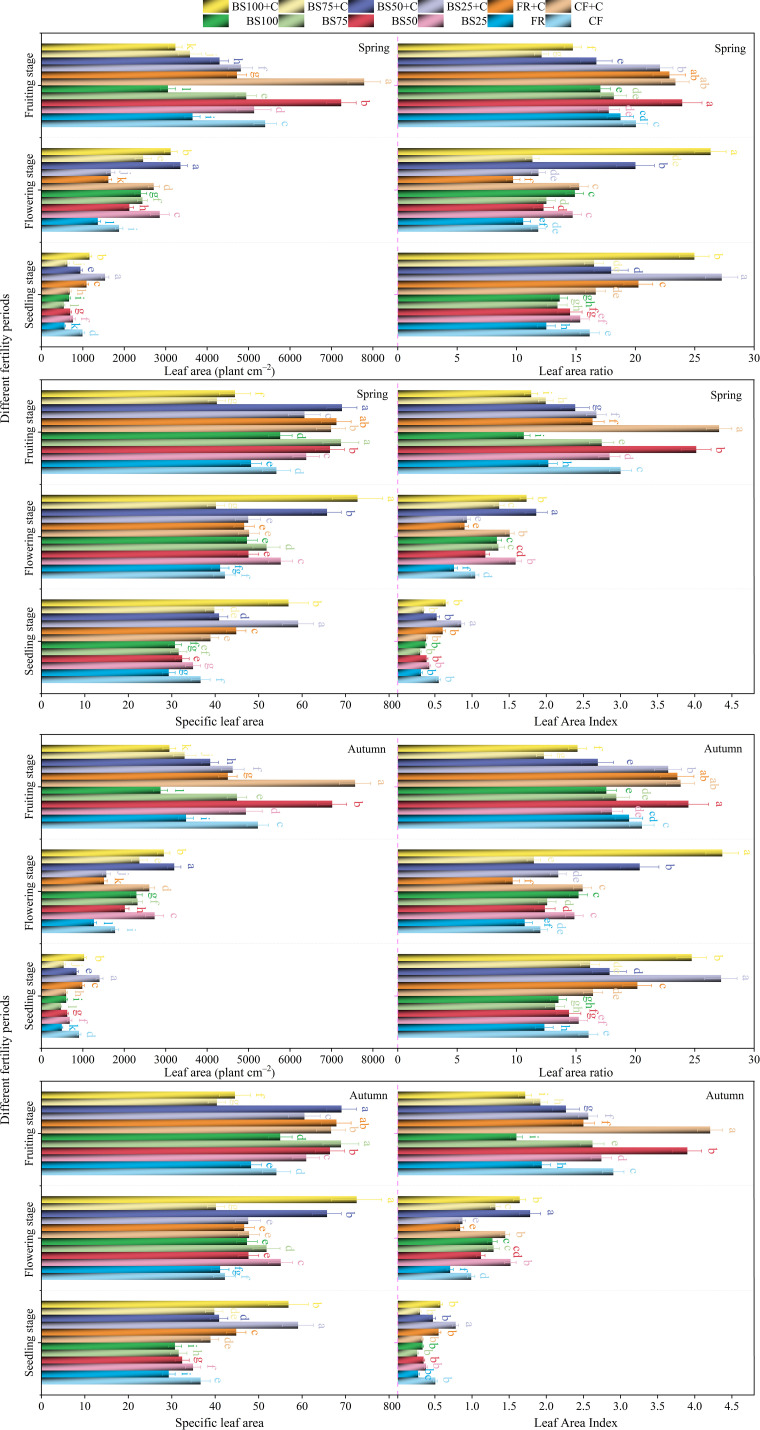
Effects of biogas slurry combined with biochar as chemical fertilizer replacements on morphological indicators of greenhouse tomatoes. Different letters within the same growth stage denote statistically significant differences between treatments at the 5% level (*P* < 0.05), and this notation is consistently applied across all subsequent figures. The error bars in the bar chart represent the mean ± standard deviation (SD) calculated from three replicates.

Under the same substitution ratio, biochar application contributed to an increase in leaf area (LA), leaf area ratio (LAR), specific leaf area (SLA), and leaf area index (LAI) of greenhouse tomatoes (**Figure 3**). From the seedling stage to the fruiting stage, LA exhibited a gradual upward trend, reaching the maximum across all treatments at the fruiting stage. The highest LA values were recorded under BS25+C (1,537.62 cm^2^ in spring and 1,404.12 cm^2^ in autumn) at seedling stage, BS50+C (3,355.57 cm^2^ in spring and 3,207.05 cm^2^ in autumn) at flowering stage, and CF+C (7,788.06 cm^2^ in spring and 7,570.78 cm^2^ in autumn) at fruiting stage (**Figures 3A, E**). LAR followed a “decrease-increase” trend under all treatments except for BS50+C and BS100+C, which exhibited an “increase-decrease” pattern. BS100+C showed the highest LAR at seedling stage and flowering stage, while CF+C had the highest LAR at fruiting stage, increasing by 54.51%, 122.72%, and 16.58% (spring), and 54.08%, 127.63%, and 15.81% (autumn), respectively, compared to CF (**Figures 3B, F**). The highest SLA values at seedling, flowering, and fruiting stages were recorded under BS25+C (59.07 cm^2^ g^−1^ in spring and 59.06 cm^2^ g^−1^ in autumn), BS100+C (72.74 cm^2^ g^−1^ in spring and 72.53 cm^2^ g^−1^ in autumn), and BS50+C (69.16 cm^2^ g^−1^ in spring and 69.06 cm^2^ g^−1^ in autumn), which were significantly higher than under CF by 61.24%, 72.28%, and 27.91% (spring), and 61.21%, 71.80%, and 27.72% (autumn), respectively (**Figures 3C, G**). LAI ranged between 0.30–0.85 at seedling stage, 0.76–1.86 at flowering stage, and 1.70–4.33 at fruiting stage in spring (**Figure 3D**), and 0.26–0.78, 0.71–1.78, and 1.60–4.21 in autumn (**Figure 3H**).

Tomato fruit yield across all treatments in descending order was as follows: 151,341.0 kg ha^−1^ under BS75+C, 141,402.0 kg ha^−1^ under BS25, 139,950.0 kg ha^−1^ under BS100+C, 137,829.0 kg ha^−1^ under FR+C, 134,037.0 kg ha^−1^ under BS25+C, 132,274.5 kg ha^−1^ under BS50+C, 130,416.0 kg ha^−1^ under CF+C, 129,960.0 kg ha^−1^ under BS75, 129,706.5 kg ha^−1^ under BS50, 124,770.0 kg ha^−1^ under BS100, 118,164.0 kg ha^−1^ under CF, and 101,380.5 kg ha^−1^ under FR. BS75+C resulted in 7.03%–49.28% higher tomato fruit yield than the other treatments. The results indicated that BS75+C significantly promoted plant height and stem diameter of greenhouse tomatoes. LA, LAR, SLA, and LAI progressively increased throughout the entire growth period, reaching their highest values at the fruiting stage. Additionally, BS75+C treatment resulted in the highest tomato fruit yield, followed by BS25 treatment.

#### 3.1.2 Tomato growth parameters

**Figure 4:**
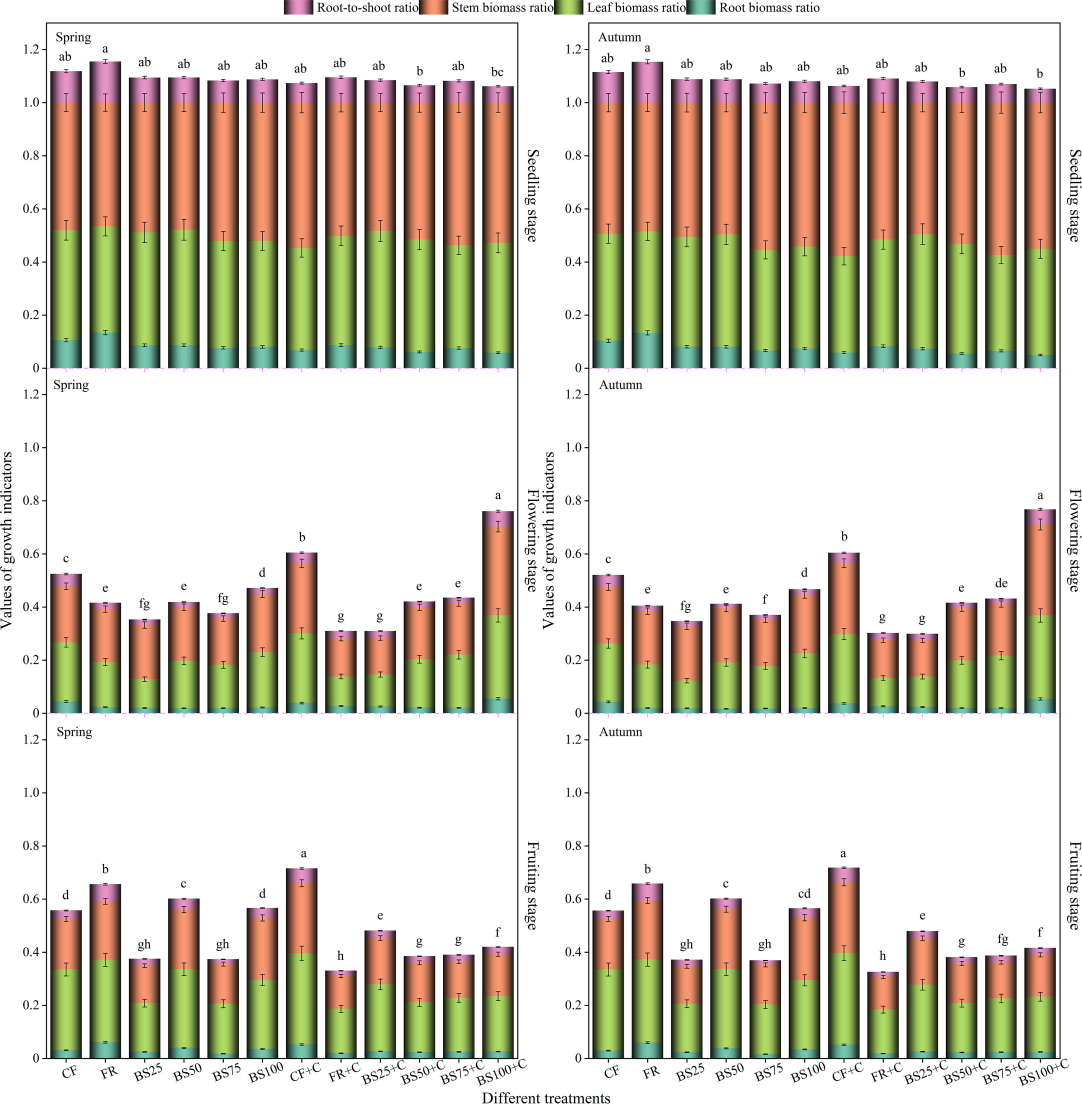
Effects of biogas slurry combined with biochar as chemical fertilizer replacements on growth indicators of greenhouse tomatoes. Treatments labeled with different letters are significantly different at the 5% level (P < 0.05). The error bars in the bar chart represent the mean ± standard deviation (SD) calculated from three replicates.

The optimal treatments for root biomass ratio (RBR), stem biomass ratio (SBR), leaf biomass ratio (LBR), and root-to-shoot ratio (RSR) varied at seedling stage (**Figures 4A, D**). At flowering stage, BS100+C treatment resulted in the highest RBR, SBR, LBR, and RSR, which increased significantly by 23.63%, 58.25%, 40.97%, and 25.00% compared to CF in spring, reaching 0.055, 0.334, 0.314, and 0.058, respectively (**Figure 4B**), and by 24.79%, 60.86%, 43.24%, and 26.20% compared to CF in autumn, reaching 0.054, 0.342, 0.315, and 0.057, respectively (**Figure 4E**). At fruiting stage of tomatoes, FR treatment showed the highest RBR, LBR, and RSR, with 96.67%, 2.09%, and 102.92% increases compared to CF in spring, reaching 0.061, 0.311, and 0.065, respectively (**Figure 4C**), and 101.99%, 2.37%, and 108.48% increases compared to CF in autumn, reaching 0.060, 0.313, and 0.064, respectively (**Figure 4F**). These results indicated that, under equal irrigation conditions, soil testing-based fertilization (FR) treatment outperformed the traditional fertilization control (CF) treatment.

Throughout the entire tomato growing stages, both RBR and RSR under CF, BS75, FR+C, and BS100+C treatments showed a decreasing trend as plant growth stage progressed, whereas the other treatments followed a “decrease-increase” pattern. LBR under BS100+C exhibited a continuous decrease from seedling stage to fruiting stage, while the other treatments followed a “decrease-increase” trend. SBR exhibited the opposite trend compared to RBR, LBR, and RSR. Except for all FR, BS50, BS100, and BS25+C treatments, which followed a “decrease-increase” pattern, SBR showed a continuous decreasing trend throughout the entire growing stages. This suggested that an appropriate biogas slurry and biochar substitution for chemical fertilizer at flowering and fruiting stages had a significant impact on greenhouse tomato growth indicators. Most growth indicators exhibited a “high-low-high” trend, and generally reached their highest values at seedling stage.

#### 3.1.3 Root morphology and root vitality

Root morphological parameters and root vitality (RA) are critical indicators for evaluating tomato root growth. Under equal N-P_2_O_5_-K_2_O input conditions, the highest RA was obtained under BS75+C treatment (**Tables 2**, **3**). RA followed an “increase-decrease” pattern, peaking at flowering stage before declining at fruiting stage. Compared to CF, BS75+C increased RA by 187.66%, 42.23%, and 72.56% in spring, and by 200.06%, 42.82%, and 74.34% in autumn at seedling, flowering, and fruiting stages, respectively. At seedling stage, the highest root morphological parameters (RL, total root length; RV, total root volume; RSA, total root surface area) were recorded under CF, reaching 1,722.76 cm, 12.44 cm^3^, and 518.84 cm^2^ in spring, and 1,680.63 cm, 10.31 cm^3^, and 466.56 cm² in autumn, respectively. At flowering stage, FR+C treatment resulted in the highest RL, RT (number of root tips), BN (number of branch points), and RN (number of root nodules), reaching 2,863.96 cm, 3,193, 4,300, and 131 in spring, and 2,824.55 cm, 3,140, 4,263, and 129 in autumn, respectively. Meanwhile, RAD (average root diameter), RSA, and RV were highest under BS100+C, reaching 1.738 mm, 1,483.41 cm^2^, and 64.45 cm^3^ in spring, and 1.643 mm, 1,381.95 cm^2^, and 56.76 cm^3^ in autumn, respectively. At fruiting stage, BS75+C resulted in the highest root morphological parameters, with RL of 4,907.99 cm, RSA of 2,965.49 cm^2^, RV of 148.20 cm^3^, RT of 17,620, and BN of 11,082 in spring, and RL of 4,869.74 cm, RSA of 2,911.41 cm^2^, RV of 138.58 cm^3^, RT of 17,567, and BN of 11,045 in autumn. These results indicated that, compared to traditional fertilization control (CF), BS75+C treatment significantly enhanced RA (*P* < 0.05), with the highest RA observed at flowering stage. Additionally, root morphology at seedling stage was primarily influenced by chemical fertilizer application, whereas biochar had a more pronounced impact on root morphology at flowering and fruiting stages.

**Table 2:** Effects of biogas slurry combined with biochar as chemical fertilizer replacements on the root characteristics of spring greenhouse tomatoes.

Period of duration	Treatments	Total root length (cm)	Average root diameter (mm)	Total root surface area (cm²)	Total root volume (cm³)	Number of root tips	Number of branch points	Number of root nodules	Root vitality (μg g^−1^ h^−1^)
Seedling stage	CF	1722.76 ± 4.93a	0.959 ± 0.02b	518.84 ± 3.79a	12.44 ± 0.58a	1449 ± 13.11c	2034 ± 36.55c	33 ± 0.58d	56.79 ± 0.68k
	FR	1290.92 ± 1.53c	0.801 ± 0.02bc	324.65 ± 3.60d	6.50 ± 0.36b	2482 ± 8.74b	2270 ± 42.16b	51 ± 1.23c	49.58 ± 0.52l
	BS25	695.56 ± 6.81h	1.345 ± 0.03a	293.83 ± 6.66e	9.88 ± 0.42ab	690 ± 12.29l	578 ± 15.36j	56 ± 1.05a	91.11 ± 0.74f
	BS50	816.64 ± 2.36f	1.074 ± 0.01ab	275.42 ± 4.04f	7.39 ± 0.39b	792 ± 9.64j	639 ± 20.18i	26 ± 0.62e	84.94 ± 0.63g
	BS75	632.86 ± 8.61i	0.565 ± 0cd	112.2 ± 8.83i	1.58 ± 0.08cd	727 ± 13.45k	522 ± 12.34k	10 ± 0.04h	94.45 ± 0.26e
	BS100	1051.73 ± 0.76e	0.835 ± 0.01bc	275.70 ± 3.79f	5.75 ± 0.21b	1189 ± 3.79e	1082 ± 5.69e	12 ± 0.21g	69.27 ± 0.67i
	CF+C	608.79 ± 7.26j	0.592 ± 0.01	113.19 ± 6.11i	1.68 ± 0.14cd	848 ± 12.00i	694 ± 29.67h	14 ± 0.35f	63.36 ± 0.42j
	FR+C	1638.52 ± 2.36b	0.759 ± 0.04c	390.61 ± 4.93b	7.41 ± 0.48b	2760 ± 10.07a	2675 ± 60.59a	53 ± 2.35b	123.06 ± 0.85c
	BS25+C	783.66 ± 5.22g	0.757 ± 0.01c	186.26 ± 5.22g	3.52 ± 0.15c	1056 ± 10.98f	938 ± 36.58f	6 ± 0.28j	99.03 ± 0.77d
	BS50+C	1251.46 ± 7.81d	0.755 ± 0.01c	296.81 ± 8.50e	5.60 ± 0.13bc	1312 ± 14.53d	1299 ± 42.16d	8 ± 0.06i	135.51 ± 1.45b
	BS75+C	602.55 ± 1.47j	0.72 ± 0c	136.21 ± 2.82h	2.45 ± 0.20c	953 ± 12.21g	795 ± 20.96g	2 ± 0l	163.36 ± 0.96a
	BS100+C	810.37 ± 3.41f	1.388 ± 0.03a	353.22 ± 4.41c	12.26 ± 0.61a	921 ± 16.72h	781 ± 10.24g	4 ± 0k	77.73 ± 1.24h
Flowering stage	CF	2602.71 ± 6.25d	1.568 ± 0.06b	1281.16 ± 4.23b	50.21 ± 1.64b	2013 ± 15.21e	2321 ± 30.25c	46 ± 0.54c	252.37 ± 2.46k
	FR	2176.27 ± 5.13j	1.028 ± 0.02d	702.29 ± 4.36i	18.04 ± 0.52g	1954 ± 12.46f	2032 ± 20.63e	14 ± 0.16h	245.16 ± 3.52l
	BS25	2234.59 ± 4.62i	1.17 ± 0.02d	820.89 ± 5.89h	24.01 ± 0.36f	2179 ± 10.58d	2244 ± 45.26d	16 ± 0.03g	286.69 ± 0.56f
	BS50	1880.39 ± 5.67l	1.435 ± 0.03bc	847.08 ± 3.27g	30.38 ± 0.40e	1382 ± 16.23j	1341 ± 50.23j	29 ± 0.45e	280.52 ± 4.52g
	BS75	2404.82 ± 7.95h	1.438 ± 0.03bc	1085.81 ± 6.24d	39.03 ± 0.28c	1430 ± 10.45i	1802 ± 59.64f	35 ± 0.55d	290.03 ± 5.24e
	BS100	2455.63 ± 9.64g	1.146 ± 0.03bc	883.89 ± 5.46f	25.33 ± 0.53f	2585 ± 17.42b	2638 ± 36.58b	22 ± 0.01f	264.85 ± 3.23i
	CF+C	2628.87 ± 8.26c	1.384 ± 0.02c	1142.23 ± 3.94c	39.51 ± 0.74c	2570 ± 16.87b	2655 ± 58.23b	81 ± 0.67b	258.94 ± 5.14j
	FR+C	2863.96 ± 10.75a	1.272 ± 0.02cd	1143.46 ± 8.93c	36.35 ± 0.28d	3193 ± 17.86a	4300 ± 84.26a	131 ± 3.62a	318.64 ± 2.18c
	BS25+C	2573.83 ± 2.34e	1.584 ± 0.01ab	1280.04 ± 7.69b	50.68 ± 0.97b	1689 ± 5.43h	1602 ± 32.16i	29 ± 0.53e	294.61 ± 8.65d
	BS50+C	2132.15 ± 3.21k	1.448 ± 0.04bc	969.06 ± 6.45e	35.06 ± 0.65d	1872 ± 9.26g	1700 ± 29.59g	4 ± 0i	331.09 ± 6.92b
	BS75+C	2477.82 ± 1.59f	1.254 ± 0d	975.68 ± 6.02e	30.59 ± 0.59e	2303 ± 3.87c	1797 ± 16.30f	4 ± 0i	358.94 ± 4.58a
	BS100+C	2718.11 ± 4.62b	1.738 ± 0.05a	1483.41 ± 9.41a	64.45 ± 2.43a	2012 ± 14.23e	1653 ± 28.45h	15 ± 0.05gh	273.31 ± 3.26h
Fruiting stage	CF	3236.43 ± 6.53k	1.941 ± 0.05ab	1972.07 ± 8.62j	95.67 ± 3.21h	7993 ± 18.63i	5485 ± 56.28k	242 ± 5.24h	146.87 ± 2.14k
	FR	3356.00 ± 6.49j	1.842 ± 0.08b	1941.02 ± 7.69k	89.38 ± 2.68i	10347 ± 35.69d	8330 ± 62.32c	533 ± 9.12a	139.66 ± 1.89l
	BS25	3505.46 ± 8.69i	1.921 ± 0.04ab	2114.20 ± 9.41i	101.52 ± 4.53f	9207 ± 29.62f	6365 ± 65.87e	245 ± 4.56g	181.19 ± 2.36f
	BS50	3685.43 ± 7.26h	1.915 ± 0.06ab	2215.80 ± 9.56g	106.06 ± 4.55e	8882 ± 59.63g	5975 ± 46.23h	184 ± 3.28i	175.02 ± 0.52g
	BS75	3898.10 ± 9.47e	1.953 ± 0.03ab	2390.30 ± 10.46e	116.69 ± 5.24d	8286 ± 45.26h	5918 ± 68.49i	170 ± 5.43j	184.53 ± 1.35e
	BS100	3980.05 ± 8.61d	2.005 ± 0.10a	2505.79 ± 9.87d	125.60 ± 3.16c	7030 ± 39.81k	5658 ± 89.25j	288 ± 4.25e	159.35 ± 0.87i
	CF+C	3741.00 ± 10.98g	1.842 ± 0.08b	2163.69 ± 11.26h	99.63 ± 5.27g	13347 ± 79.58b	8330 ± 56.27c	533 ± 10.32a	153.44 ± 2.27j
	FR+C	3780.75 ± 12.43f	1.981 ± 0.05ab	2351.46 ± 11.34f	116.44 ± 1.30d	7711 ± 96.32j	6059 ± 68.42g	439 ± 7.24d	213.14 ± 4.51c
	BS25+C	3025.92 ± 11.29l	1.822 ± 0.03b	1731.20 ± 8.62l	78.85 ± 4.36j	11219 ± 88.49c	6207 ± 85.39f	111 ± 5.22k	189.11 ± 3.48d
	BS50+C	4724.38 ± 14.56b	1.836 ± 0.02b	2829.99 ± 15.43b	129.92 ± 5.22b	9427 ± 75.34e	8671 ± 76.12b	272 ± 1.26f	225.59 ± 6.25b
	BS75+C	4907.99 ± 16.74a	1.999 ± 0.06ab	2965.49 ± 14.95a	148.20 ± 6.13a	17620 ± 98.45a	11082 ± 150.24a	481 ± 5.48c	253.44 ± 1.76a
	BS100+C	4028.73 ± 13.58c	2.030 ± 0.08a	2567.55 ± 12.40c	130.27 ± 1.48b	6435 ± 56.30l	6228 ± 86.06e	510 ± 6.49b	167.81 ± 0.24h

Data are shown as mean ± standard deviation (*n* = 3). Within each column, values followed by different lowercase letters indicate statistically significant differences at the 5% level (*P* < 0.05).

**Table 3:** Effects of biogas slurry combined with biochar as chemical fertilizer replacements on the root characteristics of autumn greenhouse tomatoes.

Period of duration	Treatments	Total root length (cm)	Average root diameter (mm)	Total root surface area (cm²)	Total root volume (cm³)	Number of root tips	Number of branch points	Number of root nodules	Root vitality (μg g^−1^ h^−1^)
Seedling stage	CF	1680.63 ± 8.24a	0.884 ± 0.03ab	466.56 ± 3.52a	10.31 ± 0.46a	1396 ± 10.57c	1997 ± 29.86c	31 ± 0.46d	53.27 ± 0.62k
	FR	1248.78 ± 6.38c	0.706 ± 0.02ab	276.80 ± 1.24d	4.88 ± 0.13cd	2429 ± 12.24b	2233 ± 34.15b	49 ± 0.52c	46.06 ± 0.54l
	BS25	653.43 ± 2.09i	1.250 ± 0.04a	256.51 ± 0.98e	8.02 ± 0.22b	637 ± 6.48l	541 ± 10.29k	54 ± 0.51a	87.59 ± 0.67f
	BS50	774.51 ± 3.41f	0.979 ± 0.03ab	238.09 ± 1.02g	5.83 ± 0.34c	739 ± 7.02j	602 ± 11.43j	24 ± 0.24e	81.42 ± 0.61g
	BS75	590.73 ± 2.14j	0.470 ± 0.01bc	87.11 ± 0.67k	1.02 ± 0.05e	674 ± 6.51k	485 ± 6.79l	8 ± 0.06h	90.93 ± 0.75e
	BS100	1009.60 ± 4.52e	0.740 ± 0.02ab	234.53 ± 0.95h	4.34 ± 0.31cd	1136 ± 9.85e	1045 ± 19.84e	10 ± 0.12g	65.75 ± 0.48i
	CF+C	566.66 ± 1.79k	0.497 ± 0.01bc	88.45 ± 1.36k	1.10 ± 0.06e	795 ± 7.24i	657 ± 12.45i	12 ± 0.10f	59.84 ± 0.57j
	FR+C	1596.39 ± 7.65b	0.664 ± 0.01b	332.94 ± 3.29b	5.53 ± 0.29cd	2707 ± 12.63a	2638 ± 30.18a	51 ± 0.49b	119.54 ± 1.26c
	BS25+C	741.53 ± 3.54h	0.662 ± 0.02b	154.12 ± 2.08i	2.55 ± 0.41e	1003 ± 8.47f	901 ± 4.26f	4 ± 0.05k	95.51 ± 0.82d
	BS50+C	1209.33 ± 6.55d	0.660 ± 0.02b	250.74 ± 2.51f	4.14 ± 0.26d	1259 ± 11.09d	1262 ± 20.47d	6 ± 0.05i	131.99 ± 1.35b
	BS75+C	560.42 ± 2.63l	0.625 ± 0.01b	109.96 ± 1.47j	1.72 ± 0.12e	900 ± 8.43g	758 ± 15.40g	5 ± 0.03j	159.84 ± 1.42a
	BS100+C	768.24 ± 3.76g	1.293 ± 0.03a	311.91 ± 4.03c	10.08 ± 0.51a	868 ± 8.06h	744 ± 6.21h	2 ± 0.03l	74.21 ± 0.44h
Flowering stage	CF	2563.30 ± 18.95d	1.473 ± 0.06ab	1185.26 ± 6.29b	43.64 ± 1.38b	1960 ± 10.86f	2284 ± 29.17d	44 ± 0.56c	248.85 ± 3.41k
	FR	2136.86 ± 13.26j	0.933 ± 0.03c	625.82 ± 3.58k	14.59 ± 0.47g	1901 ± 9.58g	1995 ± 12.35f	13 ± 0.09j	241.64 ± 2.79l
	BS25	2195.18 ± 13.43i	1.075 ± 0.05c	740.91 ± 4.62j	19.91 ± 0.52f	2126 ± 13.47e	2207 ± 20.36e	14 ± 0.14j	283.17 ± 4.52f
	BS50	1840.98 ± 9.64l	1.340 ± 0.06b	774.38 ± 4.74i	25.93 ± 0.49e	1329 ± 7.25k	1304 ± 9.58l	27 ± 0.23g	277.00 ± 4.06g
	BS75	2365.41 ± 15.42h	1.343 ± 0.06b	997.43 ± 5.89e	33.49 ± 0.60c	1377 ± 7.56j	1765 ± 11.32g	33 ± 0.48e	286.51 ± 4.65e
	BS100	2416.22 ± 17.18g	1.051 ± 0.05c	797.61 ± 5.21h	20.96 ± 0.27f	2532 ± 14.11b	2601 ± 23.14c	20 ± 0.16i	261.33 ± 1.49i
	CF+C	2589.46 ± 18.06c	1.289 ± 0.05b	1047.83 ± 7.15c	33.76 ± 0.63c	2517 ± 13.87c	2618 ± 23.37b	79 ± 1.58b	255.42 ± 1.25j
	FR+C	2824.55 ± 17.14a	1.177 ± 0.02bc	1043.45 ± 6.92d	30.69 ± 0.15d	3140 ± 24.19a	4263 ± 30.59a	129 ± 2.02a	315.12 ± 6.01c
	BS25+C	2534.42 ± 6.43e	1.489 ± 0.04ab	1184.80 ± 7.22b	44.10 ± 0.86b	1636 ± 8.25i	1565 ± 10.55k	27 ± 0.63f	291.09 ± 5.47d
	BS50+C	2092.74 ± 11.09k	1.352 ± 0.03b	888.69 ± 5.08f	30.05 ± 0.72d	1819 ± 9.43h	1663 ± 10.78i	25 ± 0.24h	327.57 ± 6.22b
	BS75+C	2438.41 ± 17.10f	1.159 ± 0.01bc	887.40 ± 2.47g	25.71 ± 0.24e	2250 ± 12.64d	1760 ± 5.64h	38 ± 0.50d	355.42 ± 5.73a
	BS100+C	2678.70 ± 12.55b	1.643 ± 0.02a	1381.95 ± 6.71a	56.76 ± 1.50a	1959 ± 9.02f	1616 ± 7.62j	13 ± 0.12j	269.79 ± 2.04h
Fruiting stage	CF	3198.18 ± 7.02k	1.846 ± 0.04b	1853.30 ± 8.47j	85.51 ± 3.30i	7940 ± 18.25i	5448 ± 48.37l	214 ± 7.38k	143.35 ± 4.36j
	FR	3317.75 ± 8.14j	1.747 ± 0.03bc	1819.88 ± 7.54k	79.48 ± 2.54j	10294 ± 37.34d	8293 ± 60.92d	440 ± 10.02f	136.14 ± 3.27k
	BS25	3467.21 ± 7.26i	1.826 ± 0.06b	1987.65 ± 9.08i	90.72 ± 4.63g	9154 ± 30.28f	6328 ± 54.17e	485 ± 9.43c	181.09 ± 4.52e
	BS50	3647.18 ± 7.58h	1.820 ± 0.06b	2083.95 ± 9.23g	94.80 ± 4.78f	8829 ± 29.06g	5938 ± 52.08i	265 ± 5.62i	181.01 ± 2.09e
	BS75	3859.85 ± 6.13e	1.858 ± 0.05b	2251.64 ± 10.36e	104.58 ± 5.36e	8233 ± 20.15h	5881 ± 28.14j	251 ± 5.08j	171.50 ± 5.43f
	BS100	3941.81 ± 10.22d	1.910 ± 0.09ab	2364.06 ± 9.75d	112.88 ± 7.81c	6977 ± 40.38k	5621 ± 36.49k	206 ± 4.83l	155.83 ± 3.46h
	CF+C	3702.75 ± 8.36g	1.747 ± 0.03bc	2031.06 ± 11.21h	88.70 ± 3.52h	13294 ± 62.14b	8293 ± 62.02c	308 ± 6.25h	149.92 ± 2.19i
	FR+C	3742.50 ± 8.41f	1.886 ± 0.05b	2215.97 ± 11.29f	104.47 ± 5.28e	7658 ± 28.10j	6022 ± 40.18h	445 ± 7.09e	209.62 ± 6.72c
	BS25+C	2987.67 ± 4.58l	1.727 ± 0.02bc	1620.15 ± 8.53l	69.95 ± 6.03k	11166 ± 79.26c	6170 ± 70.48g	523 ± 9.64b	185.59 ± 5.24d
	BS50+C	4686.13 ± 15.49b	1.741 ± 0.07bc	2562.23 ± 13.46b	111.54 ± 5.73d	9374 ± 38.15e	8634 ± 86.24b	344 ± 2.18g	222.07 ± 6.48b
	BS75+C	4869.74 ± 11.38a	1.904 ± 0.04ab	2911.41 ± 14.78a	138.58 ± 6.24a	17567 ± 85.72a	11045 ± 132.80a	548 ± 6.37a	249.92 ± 3.02a
	BS100+C	3990.48 ± 12.64c	1.935 ± 0.02a	2424.07 ± 13.02c	117.24 ± 6.08b	6382 ± 52.01l	6191 ± 68.44f	463 ± 6.41d	164.29 ± 2.19g

Data are shown as mean ± standard deviation (*n* = 3). Within each column, values followed by different lowercase letters indicate statistically significant differences at the 5% level (*P* < 0.05).

### 3.2 Comprehensive evaluation of greenhouse tomato growth quality

#### 3.2.1 Selection of evaluation indicators

**Figure 5:**
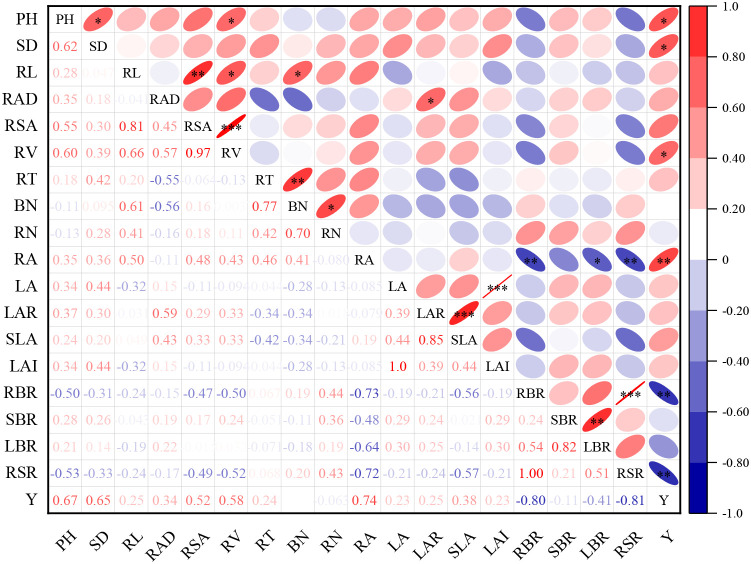
Correlation between plant growth parameters and greenhouse tomato yield. Statistical significance is indicated by *, **, and *** for *P* ≤ 0.05, 0.01, and 0.001, respectively.

Under equal N-P_2_O_5_-K_2_O input conditions, correlation analysis was conducted to examine how tomato growth parameters—including morphological, biomass, and functional traits—relate to fruit yield across varying biogas slurry and biochar substitution treatments. Parameters exhibiting statistically significant relationships with yield were identified at thresholds of *P* < 0.05, *P* < 0.01, and *P* < 0.001. As shown in **Figure 5**, PH, SD, RV, RA, RBR, and RSR exhibited highly significant positive correlations with fruit yield (*P* < 0.001), indicating that these growth characteristics have substantial effects on yield formation. Therefore, these growth parameters were included in the growth quality index (GQI) system. Based on the 2024 data set, PH, SD, RV, RA, RBR, and RSR were selected as effective indicators for GQI evaluation.

#### 3.2.2 Establishment of minimum data set

This study initially selected 18 greenhouse tomato growth indicators, forming the total growth quality data set (GQI-TDS). Through correlation analysis, 6 growth indicators significantly affecting greenhouse tomato fruit yield were identified, constituting the important data set (IDS). To calculate GQI-TDS, principal component analysis (PCA) was performed, yielding the communal variances of the growth quality indicators in the TDS as follows: PH (0.837), SD (0.741), LA (0.892), LAR (0.838), SLA (0.961), LAI (0.892), RBR (0.942), SBR (0.783), LBR (0.908), RSR (0.941), RL (0.851), RAD (0.734), RSA (0.963), RV (0.973), RT (0.896), BN (0.973), RN (0.891), and RA (0.942).

To calculate GQI-MDS, PCA was performed to address potential multicollinearity among the important growth quality indicators. Based on the eigenvalue > 1 criterion, two principal components (PC1 and PC2) were identified, accounting for 79.244% of the total variance. For each extracted principal component, indicators with factor loadings in the top 10% were selected for MDS screening (**Table 4**). In PC1, RSR demonstrated the highest norm value (1.777), whereas RBR exhibited significant correlations (|*r*| > 0.5) with other indicators, despite being within the top 10% range, leading to the selection of only RSR for the MDS. However, since RBR was not only an indicator of plant adaptability but also a key parameter in studying tomato–ecosystem interactions, RBR was retained in PC1. Additionally, RV and RA, which influenced nutrient uptake and resource utilization, were also retained in PC1. PC2 contained only one parameter (SD), which was directly included in the final MDS. Consequently, MDS indicators for growth quality evaluation were RSR, RBR, RV, RA, and SD.

**Table 4:** Principal component analysis results of growth indicators and norm values.

Indicators	Principal components		Group	Communality	Norm value
	PC1	PC2			
PH	0.750	0.492	1	0.805	1.527
SD	0.599	0.622	2	0.746	1.318
RV	0.722	0.250	1	0.583	1.409
RA	0.783	–0.339	1	0.727	1.543
RBR	–0.897	0.383	1	0.951	1.766
RSR	–0.907	0.347	1	0.943	1.777
Characteristic value	3.681	1.073			
Variance contribution rate (%)	61.353	17.891			
Cumulative contribution rate (%)	61.353	79.244			

A ‘group’ refers to growth indicators that are classified into one group based on having loading values ≥ 0.5 on the same principal component (PC).

**Figure 6:**
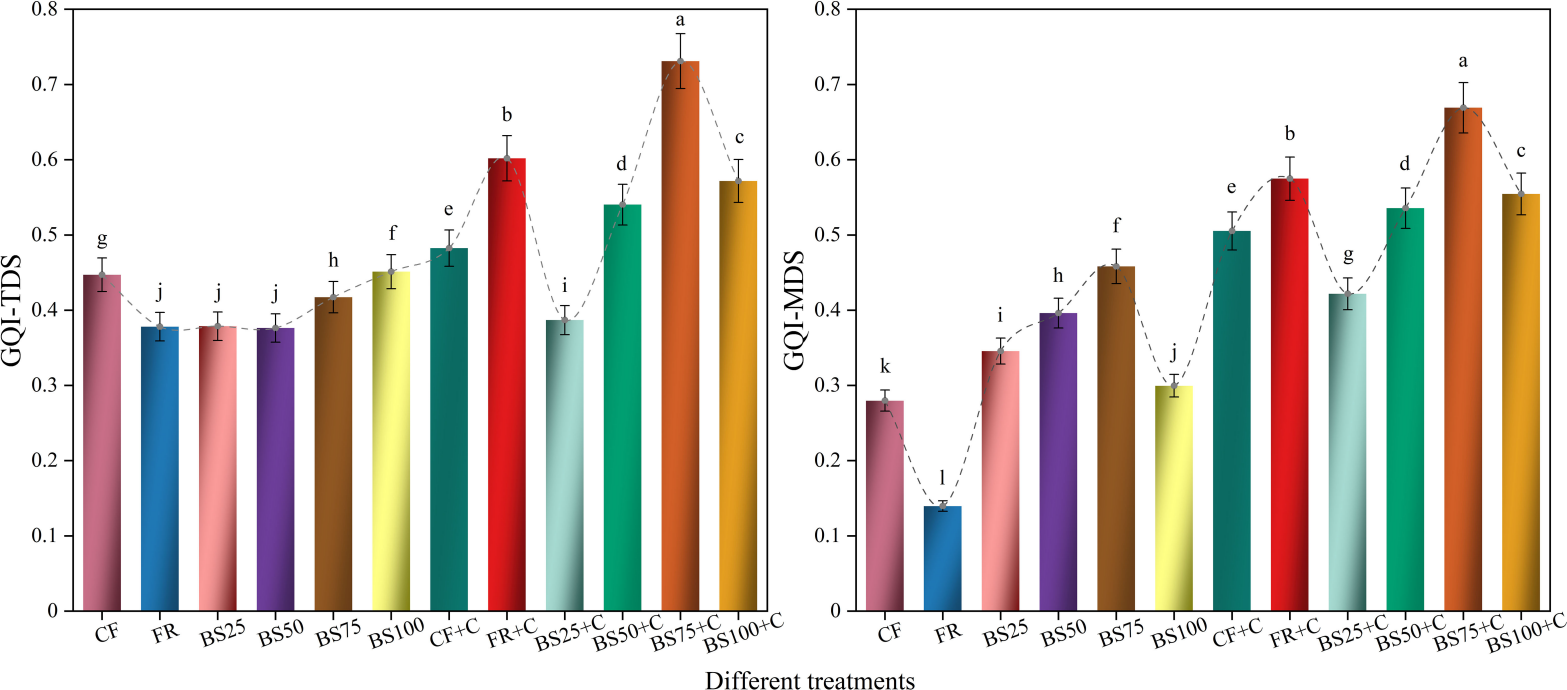
Growth quality index of greenhouse tomatoes calculated based on the total data set or minimum data set under different treatments. Treatments labeled with different letters are significantly different at the 5% level (*P* < 0.05). The error bars in the bar chart represent the mean ± standard deviation (SD) calculated from three replicates.

**Figure 7:**
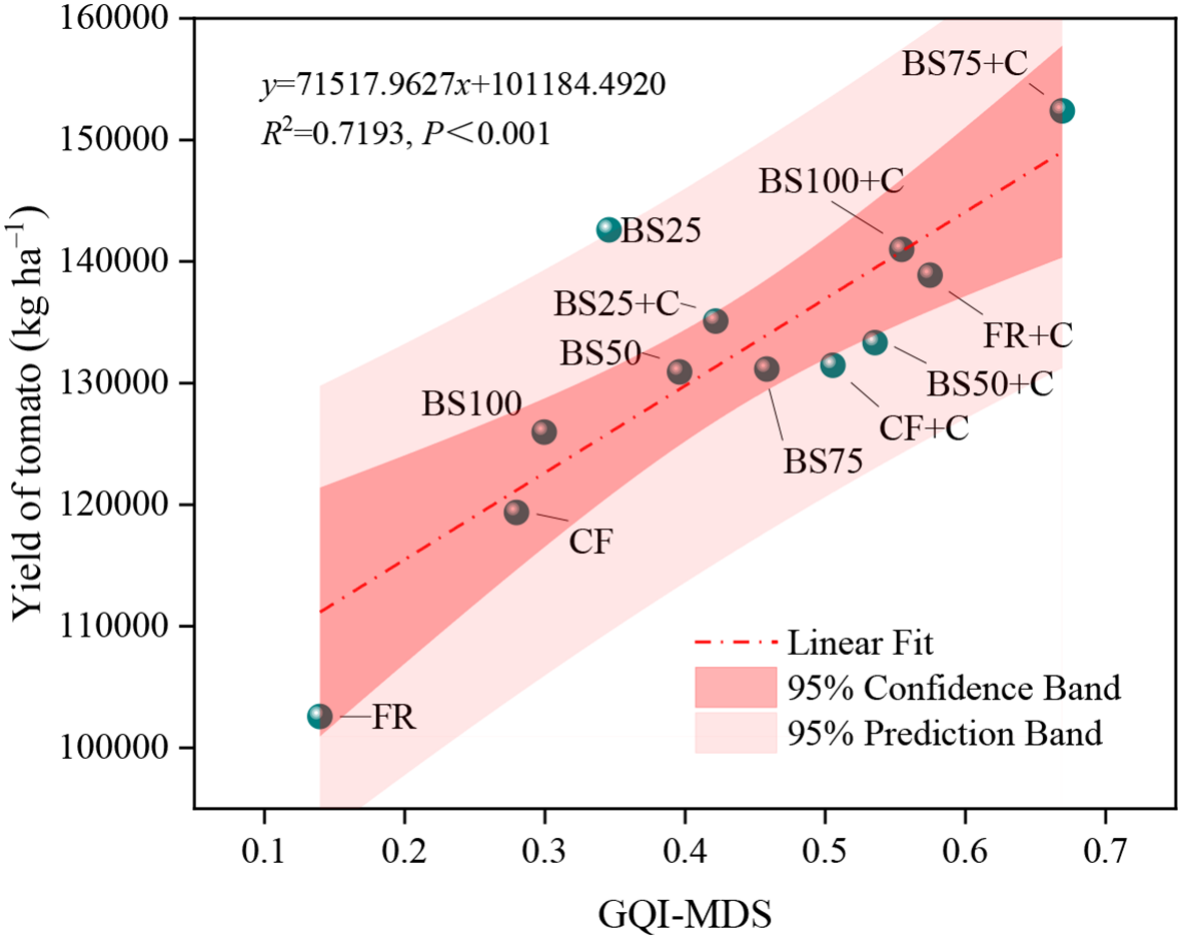
Associations between tomato yield and growth quality index under equal N, P, and K nutrient inputs and uniform irrigation conditions.

As shown in **Figure 6**, different biogas slurry and biochar substitution treatments significantly improved GQI (*P* < 0.05). The GQI-MDS ranked from the highest to the lowest, were: BS75+C (0.669), FR+C (0.575), BS100+C (0.555), BS50+C (0.536), CF+C (0.505), BS75 (0.458), BS25+C (0.422), BS50 (0.396), BS25 (0.346), BS100 (0.300), CF (0.280), and FR (0.140). The GQI under BS75+C was 16.40%, 20.65%, 24.92%, 32.38%, 46.01%, 58.61%, 68.90%, 93.52%, 123.34%, 139.14%, and 379.27% higher than the other treatments (FR+C–FR), respectively. Linear regression analysis indicated an extremely significant positive correlation (*P* < 0.001) between GQI-MDS and greenhouse tomato fruit yield, suggesting that appropriate organic-inorganic fertilization combinations contribute to improved growth quality and enhanced crop productivity (**Figure 7**).

#### 3.2.3 Validation of MDS rationality

**Figure 8:**
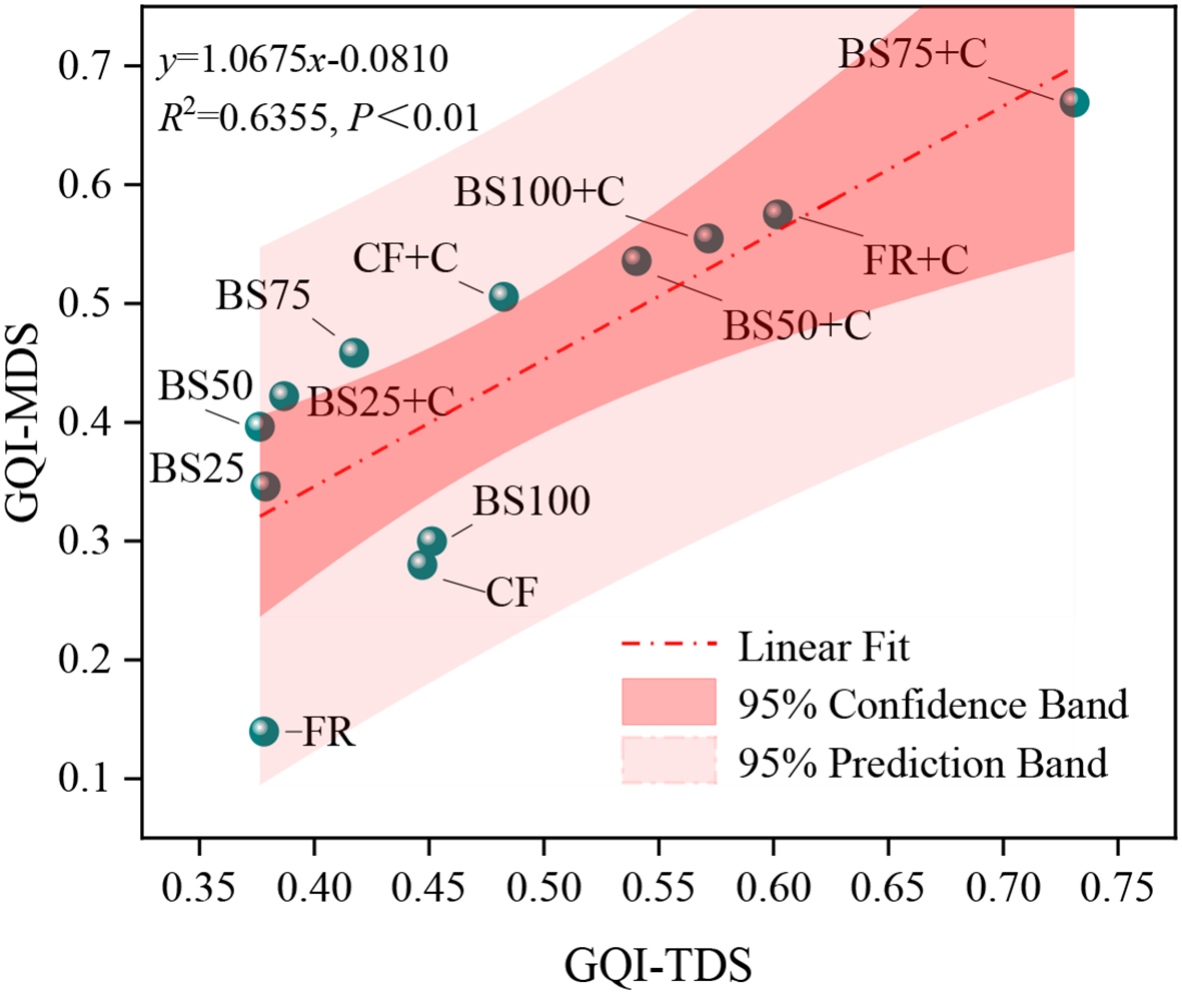
Correlation between growth quality index derived from different data sets.

Validating the MDS evaluation framework is a critical step, given that the appropriateness of indicator selection has a direct impact on the accuracy of greenhouse tomato growth quality assessments. In this study, PCA was applied to all indicators within the TDS for growth quality, determining the communal variances for each indicator. Weights were assigned based on these variances. After standardizing all indicators, they were substituted into the GQI calculation formulas to obtain the GQI values for different data sets. Results showed that GQI-TDS values ranged from 0.376 to 0.731, with an average of 0.480, while GQI-MDS values ranged from 0.140 to 0.669, with an average of 0.432. Linear regression analysis (**Figure 8**) revealed a significant positive correlation (*P* < 0.01) between GQI-TDS and GQI-MDS, with a determination coefficient (*R* ^2^) of 0.6355. These findings indicated that the MDS evaluation system developed in this study for greenhouse tomato growth quality could effectively capture the information contained in the TDS and exhibited strong representativeness.

### 3.3 Pathway analysis of GQI under different treatments

To determine the key factors affecting the variation in the growth quality index (GQI), tomato stem diameter (*Z*1), total root volume (*Z*2), root vitality (*Z*3), root biomass ratio (*Z*4), and root-to-shoot ratio (*Z*5) were selected as independent variables, and GQI (*G*) was used as the dependent variable. Variables that did not show significant effects on GQI (*P* > 0.05) were eliminated, and path analysis was subsequently conducted (**Table 5**).

**Table 5:** Path analysis results of the influencing factors of growth quality index under biogas slurry combined with biochar as chemical fertilizer replacements.

Factor	Correlation coefficient	Direct path coefficient	Indirect path coefficient					
			*Z*1→*G*	*Z*2→*G*	*Z*3→*G*	*Z*4→*G*	*Z*5→*G*	Total
SD *Z*1	0.762**	0.378		0.0832	0.1808	–1.0075	1.1276	0.3841
RV *Z*2	0.692*	0.214	0.1470		0.2172	–1.6315	1.7454	0.4781
RA *Z*3	0.781**	0.505	0.1353	0.0920		–2.3790	2.4273	0.2757
RBR *Z*4	–0.717**	3.25	–0.1172	–0.1074	–0.3697		–3.3726	–3.9669
RSR *Z*5	–0.729**	–3.376	–0.1263	–0.1106	–0.3631	3.2468		2.6468

*Zi*→*G* denotes the indirect path coefficient between the independent *Zi* and dependent variables, and *Zi* indicates the factors affecting the effectiveness of growth quality index. Statistical significance is indicated by asterisks: * for *P* ≤ 0.05, ** for *P* ≤ 0.01.

Among the direct path coefficients, root vitality (*Z*3), tomato stem diameter (*Z*1), and root biomass ratio (*Z*4) were the three major factors affecting GQI, while root-to-shoot ratio (*Z*5) had the smallest direct path coefficient (**Table 5**). Indirect path coefficient analysis revealed that among tomato morphological indicators, *Z*1 had the largest indirect effect on GQI through *Z*5, with an indirect path coefficient of 1.1276. Within functional indicators, *Z*2 and *Z*3 had the highest indirect effects on GQI through *Z*5, with indirect path coefficients of 1.7454 and 2.4273, respectively. Among growth indicators, *Z*4 exerted the greatest indirect effect on GQI through *Z*2, with an indirect path coefficient of –0.1074, while *Z*5 had the highest indirect effect on GQI through *Z*4, with an indirect path coefficient of 3.2468. Correlation analysis confirmed that *Z*3, *Z*1, and *Z*2 were the key determinants of GQI, with *Z*3 exhibiting the highest direct path coefficient (0.505). Therefore, optimizing biogas slurry-biochar substitution ratio to enhance root vitality was essential for improving GQI.

### 3.4 Multiple linear regression analysis of GQI under different treatments

In this study, multiple linear regression analysis was conducted to investigate the effects of root activity (RA), stem diameter (SD), and root biomass ratio (RBR) on the growth quality index (GQI), clarify the relative contributions of each growth parameter to GQI formation, and provide theoretical support for optimizing plant growth quality. The regression analysis indicated that the established model exhibited a good overall fit (*R* = 0.944, *R* ^2^ = 0.900, adjusted *R* ^2^ = 0.863, *P* < 0.001), explaining 90% of the variation in GQI. The regression equation (Equation 6) is as follows:


(6) GQI=0.070+0.185×RA+0.320×SD+0.346×RBR

Among the independent variables, SD showed the most significant effect on GQI (*P* = 0.029), followed by RA (*P* = 0.034), while the direct effect of RBR on GQI was not statistically significant (*P* = 0.154). The standardized regression coefficients indicated that SD (Beta = 0.559) had the greatest contribution to GQI, followed by RA (Beta = 0.369) and RBR (Beta = 0.284). These results suggest that SD and RA are the primary factors influencing GQI, while RBR may affect GQI indirectly.

Furthermore, the path analysis results demonstrated that both SD and RA exerted stable and significant positive effects on GQI. Although RBR exhibited a higher direct path coefficient, it showed a negative total effect on GQI due to its considerable negative indirect effects. The two analytical approaches were complementary to each other, revealing complex regulatory relationships among the various growth parameters and providing theoretical support for the optimization of plant growth quality.

### 3.5 Effects of different treatments on the economic benefits of greenhouse tomato production

In this study, different fertilization treatments showed marked differences in cumulative investment cost, yield revenue, and economic benefit (**Table 6**). The CF (traditional fertilization control) and FR (chemical fertilizer only) treatments had relatively low total input costs, at 6,485.58 yuan ha^−1^ and 7,912.16 yuan ha^−1^, respectively. Their corresponding yield revenues were 574,277.04 yuan ha^−1^ and 492,709.23 yuan ha^−1^, while the economic benefits reached 567,791.46 yuan ha^−1^ and 484,797.07 yuan ha^−1^, respectively. With increasing application rates of biogas slurry (from BS25 to BS100), input costs rose accordingly; however, yield revenue and economic benefit did not increase in a linear pattern. Among these treatments, BS25 performed the best, achieving a yield revenue of 687,213.72 yuan ha^−1^ and the highest economic benefit of 672,361.04 yuan ha^−1^. This indicates that applying an appropriate amount of biogas slurry is conducive to achieving an optimal cost–benefit ratio. Following the addition of biochar (+C treatments), cumulative input costs increased significantly in all treatments, exceeding 180,000 yuan ha^−1^. This rise was primarily due to the high cost of biochar. Despite the elevated costs, yield revenues improved considerably. BS75+C demonstrated the highest yield revenue at 735,517.26 yuan ha^−1^, which was significantly higher than that of BS75 without biochar (631,605.60 yuan ha^−1^). However, its economic benefit was 527,410.86 yuan ha^−1^, which remained lower than that of BS25, indicating that a higher input level does not necessarily translate into better economic returns. Similarly, BS50+C and BS100+C treatments also led to yield increases, but their economic benefits were 441,688.18 yuan ha^−1^ and 465,110.08 yuan ha^−1^, respectively, both of which were still lower than that of BS25.

**Table 6:** Effects of biogas slurry combined with biochar as chemical fertilizer replacements on the economic benefit of greenhouse tomato production.

Treatments	Chemical fertilizer cost (yuan ha^−1^)	Biogas slurry cost (yuan ha^−1^)	Biochar cost (yuan ha^−1^)	Cumulative investment cost (yuan ha^−1^)	Yield revenue (yuan ha^−1^)	Economic benefit (yuan ha^−1^)
CF	6485.58	0.00	0.00	6485.58	574277.04	567791.46
FR	7912.16	0.00	0.00	7912.16	492709.23	484797.07
BS25	7089.70	7762.97	0.00	14852.68	687213.72	672361.04
BS50	6267.25	15525.94	0.00	21793.19	630373.59	608580.40
BS75	5444.79	23288.91	0.00	28733.71	631605.60	602871.89
BS100	4622.34	31051.88	0.00	35674.22	606382.20	570707.98
CF+C	6203.53	0.00	180000.00	186203.53	633821.76	447618.23
FR+C	7630.11	0.00	180000.00	187630.11	669848.94	482218.83
BS25+C	7089.70	7135.67	180000.00	194225.37	651419.82	457194.45
BS50+C	6267.25	14898.64	180000.00	201165.89	642854.07	441688.18
BS75+C	5444.79	22661.61	180000.00	208106.40	735517.26	527410.86
BS100+C	4622.34	30424.58	180000.00	215046.92	680157.00	465110.08

In conclusion, the BS25 treatment achieved the highest economic benefit under the current experimental conditions by maintaining a moderate input level and relatively high output, making it the most cost-effective fertilization strategy. Although biochar significantly enhanced yields, particularly in the BS75+C treatment, its high cost substantially reduced net economic returns. Therefore, its application may be more suitable in long-term systems focusing on soil improvement and ecological sustainability.

## 4 Discussion

### 4.1 Effect of different biogas slurry combined with biochar as chemical fertilizer replacement on greenhouse tomato growth and fruit yield

Growth quality improvement plays a critical role in enhancing greenhouse tomato yield, and yield is also a key indicator for evaluating growth quality (Ilangumaran et al., 2022). Results of this study indicated that BS75+C treatment significantly promoted tomato plant height and stem diameter. Additionally, across all experimental treatments, LA, LAR, SLA, and LAI exhibited an increasing trend from seedling stage to fruiting stage of tomatoes, reaching the maximum values at fruiting stage, which was consistent with previous studies (Chai et al., 2023; Wu et al., 2013; Zhang et al., 2022). It is noteworthy that, under the condition of equal total nutrient input across treatments, the observed differences in yield and growth traits suggest that the regulatory mechanisms are not solely attributable to nutrient quantity, but are more likely driven by the combined effects of biochar and biogas slurry on the soil–plant system. Specifically, the highly porous structure and abundant functional groups of biochar improve soil physical properties, enhance cation exchange capacity, and increase water and nutrient retention, thereby stabilizing the rhizosphere environment and improving nutrient uptake efficiency from the slurry (Liu et al., 2016). Meanwhile, biogas slurry contains micronutrients such as Ca, Mg, Fe, and Zn, which are involved in chlorophyll synthesis and stability. These elements may also modulate plant antioxidant systems and hormonal balance, thereby enhancing photosynthetic capacity, delaying functional leaf senescence, and mitigating midday depression of photosynthesis in greenhouse-grown crops (Zheng et al., 2020; Kumar et al., 2023). Further analysis revealed a dose-dependent effect of the biochar–slurry combination on tomato growth. Under high slurry substitution ratios (e.g., BS100 or BS100+C), although leaf area ratio (LAR) was elevated during seedling to flowering stages—indicating increased investment in leaf development—leaf area (LA), specific leaf area (SLA), and leaf area index (LAI) increased less significantly, suggesting a potential imbalance favoring vegetative over root growth, or suppressed root development. These conditions may hinder the accumulation of photosynthates and ultimately affect yield formation. Prior studies have also reported that excessive slurry application can intensify ammonia volatilization, cause salt accumulation, reduce soil aeration, and lead to shoot overgrowth, leaf chlorosis, and decreased photosynthetic efficiency (Xu et al., 2013). By contrast, treatments with medium to high slurry substitution ratios (50%–75%) combined with biochar (e.g., BS50+C, BS75+C) achieved a better coordination between growth traits, canopy structure, and functional leaf efficiency. The slow-release properties of biochar and the rapid availability of nutrients in slurry complemented each other in both temporal and spatial dimensions. This synergy not only alleviated physiological stress caused by rapid nitrogen release but also improved rhizosphere pH buffering, microbial diversity, and nutrient availability, thereby enhancing soil ecological stability and crop responsiveness to external inputs (Liu et al., 2016; Du et al., 2018).

Sun et al. (2020) reported that various organic amendments, such as biochar, compost and straw, can alter the rhizosphere microbial community composition and increase root exudates (e.g., organic acids), thereby enhancing root vitality. This finding supports our study results demonstrating that compared to conventional fertilization (CF), BS75+C significantly increased RA (*P* < 0.05), with the peak at flowering stage (**Tables 2**, **3**). The primary reason may be that the biogas slurry contains abundant soluble nitrogen, phosphorus, potassium, and micronutrients, which can be directly absorbed by roots, thereby enhancing nutrient use efficiency. In addition, its low-molecular-weight organic compounds may serve as signaling substances or carbon sources to stimulate root growth (Beccaccia et al., 2015). The addition of biochar further improves soil physicochemical properties and enhances the stability of nutrient supply and slow-release capacity in the rhizosphere, thus forming a synergistic effect (Du et al., 2018). Additionally, this study found that root morphology at seedling stage was primarily influenced by the amount of chemical fertilizer applied, whereas biochar had a greater impact on root morphology at flowering and fruiting stages of tomatoes.

A preliminary analysis suggests that, at the seedling stage, the nutrient release from biogas slurry and biochar was relatively slow, and the accumulation of available nutrients in the rhizosphere had not yet reached levels sufficient for effective plant uptake. In contrast, the application of chemical fertilizers rapidly supplied soluble nitrogen, phosphorus, and potassium, thereby meeting the nutrient demands for early seedling growth (Chai et al., 2023). However, at flowering and fruiting stages, biochar combined with an appropriate biogas slurry substitution ratio optimized soil environment, promoted rapid root meristem development, and enhanced root vitality, which ultimately improved root morphology (Blackwell et al., 2010). This research showed that the combination of biogas slurry and biochar as a substitute for chemical fertilizers had a significant effect (*P* < 0.05) on greenhouse tomato growth indicators at flowering and fruiting stages, with most growth indicators exhibiting a “high-low-high” trend, reaching the highest values at seedling stage. These findings aligned with observations from Li et al. (2023). This may reflect that seedling stage represents the initial establishment phase of plant growth, during which the growth rates of various indicators tend to be uniform. Compared to conventional fertilization (CF), soil testing-based formula fertilization (FR) resulted in higher root biomass, leaf biomass, and root-to-shoot ratio (**Figure 4**). This might be because soil testing-based fertilization follows the principle of nutrient balance, allowing for scientific regulation of fertilization rates and nutrient ratios, thereby improving fertilizer use efficiency and enhancing plant growth performance.

### 4.2 Evaluation of the effect of different biogas slurry combined with biochar as chemical fertilizer replacement on greenhouse tomato growth quality

Growth quality reflects how well plants perform under specific environmental circumstances (Iost et al., 2007). Various quantitative approaches have been developed worldwide to assess growth quality, including the Nemerow composite index, grey relational analysis, TOPSIS, and the growth quality index (GQI) (Bame et al., 2014; Chang et al., 2023; Liu et al., 2025; Xie et al., 2019; Huang and Rao, 2025). Among these, GQI is particularly valuable due to its ability to integrate actual indicator values, assign appropriate weights, and reflect inter-indicator interactions. Additionally, the use of a minimum data set (MDS) helps streamline indicator selection by minimizing redundancy, while the norm value method ensures a balanced representation of each variable (Karlen et al., 2019). In this research, GQI was derived based on MDS selection and norm value standardization, allowing for a systematic evaluation of how different substitution ratios of biogas slurry and biochar influence greenhouse tomato growth. Although the indicators included in the MDS effectively represented treatment differences, selected parameters often vary across studies. For instance, Li et al. (2019) identified bulk density, cation exchange capacity, soil organic matter, alkali-dissolvable N, total K, extractable Cu, and extractable Fe as core indicators of soil quality, whereas in this study, based on 18 greenhouse tomato growth indicators, RSR, RBR, RV, RA, and SD were selected for constructing the GQI-MDS. The rationale behind these choices lies in their functional roles. RSR held the highest loading in PCA; RV and RA influenced resource uptake and utilization; RBR reflected plant adaptability under environmental stress; and SD served as a direct indicator of plant growth and development, justifying their selection in the GQI-MDS.

In this study, the BS75+C treatment produced the highest GQI value (**Figure 6**). Moreover, GQI was significantly and positively correlated with tomato fruit yield (*P* < 0.001, **Figure 7**), indicating that growth quality improvement contributed effectively to yield enhancement, which was consistent with the findings of Chang et al. (2023). To further explore the causal relationships between GQI and its MDS indicators under different biogas slurry and biochar substitution treatments, stepwise regression was applied to eliminate variables that had no significant effect on GQI, and the corresponding path analysis was conducted (**Table 5**). The results indicated that root vitality, stem diameter, and root biomass ratio were the key factors influencing GQI. This can be attributed to the fact that roots are the primary organs for water and nutrient uptake, while root vitality comprehensively reflects root absorption functions, and changes in root growth, metabolism, and vitality directly affect aboveground growth and development (Li et al., 2013; Ohnishi et al., 2010). On the other hand, stem diameter reflects nutrient accumulation and transport capacity, is closely related to the transport of photosynthetic products, structural stability, and lodging resistance, and serves as an important indicator of plant vigor (Shakoor et al., 2017). Furthermore, root biomass ratio reflects the resource allocation strategy between aboveground and belowground parts of the plant, playing a decisive role in balancing nutrient absorption and photosynthate utilization, and is a key trait for achieving high yield and quality (Lian et al., 2020). Additionally, the biomass ratios of leaves, stems, and roots reveal the resource allocation patterns among different organs during growth, and their dynamic changes are considered important adaptive mechanisms under stress environments (Poorter, 2009; Luo et al., 2023).

## 5 Conclusions

BS75+C treatment (75% biogas slurry combined with biochar as a chemical fertilizer substitute) exhibited the best enhancement effect on greenhouse tomato plant height (PH) and stem diameter (SD). Across all treatments, significantly positive effects on growth parameters were observed at flowering and fruiting stages (*P* < 0.05), with BS75+C significantly improving root activity (RA), surpassing the traditional fertilization control (CF). The growth quality index (GQI) under BS75+C treatment was the highest among all experimental treatments, and GQI was highly significantly correlated with tomato fruit yield (*P* < 0.001). Path analysis demonstrated that RA, SD, and root biomass ratio (RBR) were the primary determinants of GQI. Further multiple linear regression analysis revealed that SD (Beta = 0.559) and RA (Beta = 0.369) exerted significant direct effects on GQI, while RBR primarily influenced GQI formation through indirect pathways.

In summary, under equal nitrogen–phosphorus–potassium nutrient input (380–180–500 kg ha^–1^) and consistent irrigation conditions, the BS75+C treatment proved to be the optimal field management approach for improving greenhouse tomato yield, growth quality, and sustainable production in arid and semi-arid regions of China. Under this treatment, the average tomato yield reached 151,341.0 kg ha^–1^, providing an efficient and sustainable agricultural management solution suitable for long-term input systems aimed at soil improvement. However, the BS25 treatment achieved the highest economic benefit, reaching 672,361.04 yuan ha^–1^, based on a balance of moderate input and relatively high output.

## Data Availability

The original contributions presented in the study are included in the article/supplementary material. Further inquiries can be directed to the corresponding author.
